# Experiences with Qi and changes in post-acute sequelae of COVID-19 (PASC) symptoms with qigong: a qualitative analysis of participants’ experiences in a pilot clinical trial

**DOI:** 10.1186/s12906-025-05161-w

**Published:** 2025-11-28

**Authors:** Monica Joy, Nicole Adams, Michael Yanuck, Michelle L. Dossett

**Affiliations:** 1 University of California Davis School of Medicine, Sacramento, CA U.S.A.; 2 Department of Internal Medicine, University of California Davis, Sacramento, CA U.S.A.; 3 Sacramento VA Medical Center, Mather, CA U.S.A.; 4 Division of General Internal Medicine, 4150 V St., Suite 2400, Sacramento, CA 95817 U.S.A.

**Keywords:** Qigong, Biofield therapies, Long COVID, Post-Acute COVID-19 syndrome, Qualitative research, Traditional chinese medicine, Integrative medicine

## Abstract

**Background:**

Commonly known as “long COVID”, post-acute sequelae of COVID-19 (PASC) is a chronic condition with no validated treatment that significantly impacts the quality of life of those affected. Qigong is a Traditional Chinese Medicine (TCM) practice that may serve as a possible therapeutic approach for PASC. This study explored participants’ experiences with qi, and changes in PASC symptoms, following participation in a clinical trial of group-based, combined external and internal qigong for individuals with PASC.

**Methods:**

A qualitative study of 26 individuals who participated in a pilot feasibility trial of qigong for PASC symptoms was performed. Participants engaged in six weekly, two-hour sessions of external and internal qigong delivered in a group-based format. Upon completion of the intervention, all participants were interviewed using a semi-structured interview guide. The interviews were transcribed, coded and analyzed using a conventional content analysis approach to explore participants’ perceptions and understanding of qi, qigong, and the overall impact of the sessions on their well-being and PASC symptoms.

**Results:**

Participants’ understanding and explanations of qi varied. Almost all participants (92%) reported feeling qi during the sessions, and a variety of different sensations associated with perception of qi were described. Approximately three-quarters of participants experienced improvement in one or more PASC symptoms, most commonly fatigue, “brain fog”, and sleep quality, and 85% reported improved well-being. Additionally, participants frequently cited the group-based nature of the intervention as a positive aspect of their experience.

**Conclusions:**

The qigong intervention was well-received by participants, with the majority perceiving qi and reporting improvements in PASC-related symptoms and overall well-being. These findings suggest that many individuals may be able to perceive qi and that group-delivered combined external and internal qigong may be a beneficial complementary therapy for managing PASC symptoms. As this is a pilot study with a small sample size and a single qigong teacher, results must be interpreted cautiously. Further research is warranted to evaluate the effects of qigong in this patient population.

**Trial registration:**

Registry: ClinicalTrials.gov. Trial Registration Number: NCT05675995. Date of Registration: January 4, 2023.

## Background

It is estimated that 6.9% of United States adults, and more than 65 million people worldwide, have suffered from post-acute sequelae of COVID-19 [1, 2] (PASC, commonly known as “long COVID”), a chronic condition that can affect nearly every organ system and varies in clinical presentation [3]. PASC is defined as symptoms that persist at least 3 months following a COVID-19 infection [4]. The most common symptoms associated with PASC are fatigue/post-exertional malaise, myalgias, arthralgias, depression and anxiety, “brain fog” (i.e. problems concentrating or thinking), cough, dyspnea, and sleep disturbance. There are no current validated treatments for PASC [5], and prognosis is unknown [2]. With 44% of patients with PASC unable to work at all, and 51% reducing their working hours, PASC is responsible for an estimated $50 billion in lost income annually and increased medical spending of $9000 per person [6].

Given the burden of disability from PASC, new therapies are urgently needed. There is growing evidence that integrative medicine approaches are beneficial for many chronic health conditions, and it has been suggested that Traditional Chinese Medicine (TCM), qigong, and group-based integrative interventions may be beneficial for treating PASC [7–14]. One such therapy that has not been studied is external qigong, a biofield therapy. Biofield therapies (e.g., reiki, therapeutic touch, external qigong) are based on the premise that there is a subtle energy that permeates and surrounds the human body and can be engaged to help promote healing [15]. Despite the controversial nature of biofield therapies, some studies have shown potential benefits. For example, external qigong may reduce chronic pain [16, 17]. Qigong works with “qi,” which is considered to be “a ubiquitous resource of nature that sustains human well-being and assists in healing disease” [18]. Different forms of qigong, such as internal movement-based qigong (similar to Tai Chi) and external qigong (a form of non-touch energy healing) have been refined over the last 5000 years and are still practiced today. Qualitative research can provide a deeper understanding of the nuanced participant experience with qigong and the phenomenon of qi [19]. Existing qualitative research on qigong is limited; there are few qualitative studies characterizing patients’ experiences of qi and their understanding of it. This study aims to deepen our understanding of the PASC patient experience with a group-based combined external and internal qigong intervention, the impact of this intervention on participants’ PASC symptoms and overall well-being, and to characterize participants’ understanding and experience of qi.

## Methods

### Participant recruitment and eligibility

This qualitative study was embedded in a prospective randomized controlled trial approved by the UC Davis Institutional Review Board and registered on ClinicalTrials.gov (NCT05675995). The Standards for Reporting Qualitative Research (SRQR) were followed for this report [20]. Participants were recruited from the UC Davis Pulmonary Specialty Clinic (a clinic that serves PASC patients), UC Davis-affiliated primary care clinics and federally qualified health centers, Studypages, and social media advertising. To be eligible for inclusion in the study, participants were required to be English-speaking adults with a history of a COVID-19 infection associated with lingering symptoms lasting longer than 12 weeks. Individuals with COVID-related shortness of breath and evidence of parenchymal lung damage on imaging (or FVC < 80% on pulmonary function testing), dementia, a history of exposure to agent orange, an active pregnancy, active episode of psychosis, mania, or severe depression, active infection or substance abuse disorder(s), or any other condition that would make it difficult for the individual to complete questionnaires or attend the qigong sessions were excluded from the study. Upon determining eligibility, informed consent was obtained for all enrolled participants.

### Study design and intervention

The study intervention consisted of six weekly small group sessions of external qigong followed by a brief internal qigong practice. The qigong interventions were led by a trained practitioner (MY) who, during the external qigong practice, facilitated the release of turbid qi/retained energy of trauma with the intention of promoting healing [21]. At the first session, all participants were provided with a brief explanation and demonstration of qi (qi tangibility exercise). During the qi tangibility exercise, participants were invited to scan about six to eight inches above the practitioner’s hand and forearm to perceive points of qi. Next, participants took turns receiving the external qigong intervention as the rest of the group observed. During the intervention, participants were stationary and situated in a comfortable seated position. External qigong was conducted while the hands of the qigong practitioner were at least six inches away from the participant’s body, starting around the participant’s head and usually moving downwards. External qigong was individualized to each person, and the specific healing movement and length of treatment varied by participant and session, depending on the practitioner’s perception of the participant’s qi status. Typically, the feeling of qi left the practitioner’s hand after several minutes, so to signal the end of the intervention. After all of the participants had received the external qigong intervention, the group was invited to participate in a brief internal qigong self-practice exercise, so that the participants would have the means to release any turbid qi on their own in-between sessions [21]. Unlike most forms of internal qigong that involve movement, this practice involved minimal effort or exertion and was more like a meditation. The seated self-practice consisted of three phases: (1) breathing into the dantien (the vital energy center located about three inches below the navel and believed to be the body’s main storage area for qi), (2) feeling a qi ball between the palms for a few minutes, and (3) connecting with a feeling of the universal qi at the top of the head and feeling energy in the head and hands.

The sessions were each approximately two hours long, and group size ranged from two to nine participants. Qigong sessions were held at a primary care clinic affiliated with UC Davis on Saturday mornings when no other patients or clinic staff were present.

### Qualitative interviews

Within two weeks of completing the final group qigong session, study participants underwent a semi-structured interview regarding their experience as a participant in the study. All participants who completed the intervention were interviewed. Interviews were performed by one of two investigators (NA, MJ) who received training from an experienced qualitative researcher (MD). Interviews were audio recorded and ranged from 8 to 42 min in length. Each interview included the same series of questions, with probes to seek additional information so that participants could expand on any answers and provide additional input. The interviews queried about participant understanding and experience of qi and the practice of qigong, the impact of the group qigong sessions on their PASC symptoms and overall well-being, and their subjective experience of the study as a whole (Table 1).

**Table 1:** Interview questions

How would you describe qigong to someone who doesn’t know anything about it?
What did you feel, if anything, in terms of qi during the sessions? Did that change over time?
Did you notice any changes in your long-COVID symptoms during this study? Please explain.
Do you think the qigong sessions affected your symptoms or sense of well-being in any way? Please explain.
Is there anything else that you would like to tell us about your experience with qigong?

### Qualitative analysis

Interviews were transcribed and a conventional content analysis approach [22] was used to qualitatively analyze the semi-structured interview data in an attempt to understand participants’ experiences without any a priori guiding theory. Three team members (NA, MJ, and MD) served as the primary coders for analysis. NA is a clinical research coordinator, and MJ is a medical student. Both NA and MJ were involved in study participant recruitment and data collection and took attendance at the sessions. They were not involved in the delivery of the qigong intervention. MD is an integrative medicine researcher who has some personal experience with internal qigong and had minimal to no contact with the study participants. None of the team members had any prior experience with external qigong. During the initial phase of coding, all three study team members individually engaged in a process of iterative immersion/crystallization with the transcribed interview data, reading through portions of transcripts and pausing to reflect on participants’ experiences until patterns and themes began to emerge that could be meaningfully expressed as categories and subcategories, creating codes.

After this process was completed independently by each of the three coders with one third of the transcribed interviews, the study team met to discuss their initial set of codes and their overall impressions of the data. Based on this discussion, the team created a collective draft of codes and returned to the next one-third of transcripts, repeating the process above and meeting once again to discuss revised codes and insights. This cycle of repeated immersion/crystallization and code revision with subsequent meetings was repeated until a final set of codes was agreed upon by all three members of the study team. Using this set of codes, NA and MJ revised and reviewed their coding, re-reading transcripts and quotes to ensure that all unique participant perspectives were captured.

Based on the information provided in each interview, and blinded to the other’s coding, NA and MJ individually reviewed each transcript and coded whether participants reported experiencing an improvement in their PASC symptoms (overall and also regarding specific symptoms), overall well-being, and the extent to which they were able to perceive/feel qi. Any discrepancies in coding were resolved through the use of a “tie-breaker” in which MD made the final coding determination.

Participants were also asked to self-identify their PASC symptoms (free text response on the baseline questionnaire), and these symptoms were tallied to characterize the frequencies of the various PASC symptoms within this patient cohort.

## Results

### Demographics and baseline characteristics

Twenty-six participants completed the study and agreed to complete exit interviews which were coded and included in the analysis. These 26 participants ranged from 25 to 79 years of age; 73.1% of participants were female, 84.6% White and 11.5% Hispanic (Table 2). Average duration of PASC symptoms was 24.7 months (range 4–47). The most commonly reported PASC symptoms prior to starting the study intervention were fatigue, “brain fog” (i.e. problems concentrating or thinking), and shortness of breath (Table 3) [23].

**Table 2:** Participant demographics

Variable	Participants (*n* = 26)
Age, mean (SD), years	53.9 (14.1)
Sex	
Female, %	73.1
Male, %	26.9
Ethnicity	
Hispanic, %	11.5
Race	
White, %	84.6
Asian, %	7.7
American Indian or Alaska Native, %	3.9
Other, %	3.9
PASC symptom duration, mean (SD), months	24.7 (11.6)

**Table 3:** PASC symptoms reported by participants at baseline

PASC Symptoms	% Affected
Fatigue	88%
“Brain fog” (i.e. problems concentrating or thinking)	73%
Shortness of breath	65%
Digestive symptoms (i.e. diarrhea, stomach pain, constipation)	42%
Myalgias	42%
Headache	38%
Palpitations/tachycardia	35%
Dizziness	35%
Sleep disturbances	35%
Arthralgias	31%
Post-exertional malaise	27%
Loss of taste and/or smell	19%
Muscle weakness	15%
Memory loss	15%
Paresthesias	15%
Chest pain, tightness, or pressure	15%
Anxiety	12%
Tinnitus	12%
Vision impairment	12%
Impaired thermoregulation	8%
Tremor	8%
Rash	8%
Positional orthostatic Tachycardia	4%
Dysuria	4%
Depression	4%
Cough	4%
Changes in Menstruation	4%
Hair Loss	4%
Decreased Libido	4%

### Understanding of qigong

Participants were asked, “How would you describe qigong to someone who doesn’t know anything about it?” Their descriptions of qigong varied, sharing a few common themes. Qigong was commonly described as working with the energy of oneself, a practitioner, and other people to promote healing and to release energy blockages.

*“There are energy centers in your body and you’re trying to*,* sort of like*,* focus on the flow of energy through your body and*,* like*,* figure out where energy is being blocked and release that.” (ID 16)*.

*“I describe it to people as the Chinese energy work that has been used for many*,* many years to help equalize a person’s energy level so there’s no blockages.” (ID 45)*.

*“I would say it relates to your energy*,* and someone else stepping into your energy to kind of recharge your energy and almost fix it by just connecting with you. It’s a connection.” (ID 43)*.

Sometimes the description of qigong referenced the practitioner, Dr. Yanuck, and the explanation of qigong he provided in the first group session.

*“Let’s say like it involves a lot of energy healing and how Dr. Yanuck explained it*,* it was like releasing the retained energy of trauma*,* which made more sense like in the terms that he said.” (ID 18)*.

*“Dr. Yanuck was connecting our energy to the universal qi*,* which is like a blueprint that our body can learn from*,* essentially releasing retained energy of prior trauma*,* whether it be emotional*,* mental*,* physical*,* as to allow our system to jumpstart back into a higher functioning state. So*,* for this study specifically*,* it was addressing the multiple injuries caused by long COVID.” (ID 30)*.

Qigong was also described as a restful and calming experience, a type of Eastern medicine and self-healing that involves connecting with a universal energy.

*“For me*,* it’s been getting me into*,* like*,* this restful state where you learn to tap into the energy of your body. And the goal is to kind of have that energy that’s stored up*,* energy from trauma*,* of which COVID has been traumatic in your body*,* to try to get that trauma out of your body.” (ID 6)*.

*“A way of getting you to connect with the central energy forces within your body and with*,* you know*,* the earth.” (ID 44)*.

*“There is this energy force I knew about*,* but never really knew how to take advantage of it*,* I guess*,* or harness it in some way.” (ID 57)*.

Some participants used metaphors to explain qigong.

*“You live in a house*,* it’s just a regular house. And then suddenly*,* you open up the plank in the floor*,* and there’s a diamond*,* and it’s not just a diamond under the floor - suddenly it’s everywhere*,* and it’s beautiful*,* and it’s powerful.” (ID 23)*.

While many participants provided explanations related to energy, a few did not know how to explain qigong at all.

*“I don’t feel qualified to describe it*,* because I don’t think I actually really understand it.” (ID 14)*.

### Experience of Qi

The majority of study participants reported perceiving, or feeling, qi (92%), and their experience of qi varied across participants as well as within participants over the course of the study. Qi was often described by participants as “energy”, sometimes a ball of energy or a movement of energy felt throughout the body. Qi was perceived in themselves, in other study participants, and in the instructor, and was often felt most strongly in their own hands.

*“I felt the warmth*,* like a ball. … Ball of energy. And that was what I felt the first time. But then the subsequent sessions*,* I felt that energy beyond the ball and more*,* you know*,* throughout my body*,* radiating through my body. And I could feel the energy from other participants at times. Yeah*,* it was just… It was really bizarre*,* and it was a good feeling.” (ID 44)*.

Descriptions of the feeling of qi included temperature changes (warmth, coolness), a breeze, tingling, static or electricity, pressure or heaviness, and magnetism.

*“The sensations that I felt were more of like cool air*,* you know*,* coming from my hands in particular*,* and my arm*,* I did feel that. I felt a magnetism sensation*,* and I did feel some tingling on my crown*,* you know*,* on the top of my head.” (ID 49)*.

*“As we practiced each week*,* I started to feel the cold … And it’s fascinating because you think it’s coming from the air on the ceiling*,* but it’s really*,* what you’re feeling is in your hands … Not knowing what to expect and then actually feeling the sensations was incredible. " (ID 10)*.

*“During the sessions*,* I had a feeling of warmth*,* relaxation and tingling.” (ID 39)*.

Some participants experienced physical sensations, a release of tension, and/or emotional experiences.

*“We would experience a lot of physical symptoms that were interesting*,* such as our heart rates fluttering or*,* I know at one time I got a little sweaty or*,* you know*,* I would become hot. And*,* I don’t know*,* it’s a… I guess it was a physical manifestation of the qigong*,* is feeling it during the sessions.” (ID 12)*.

Participants described the experience of qi as comfortable, non-painful, and relaxing.

*“It was overall a kind of sense of overall relaxation*,* very pleasant*,* and did not feel uncomfortable.” (ID 50)*.

### Effect of qigong on PASC symptoms

When participants were asked how their PASC symptoms changed throughout the course of the study, 73% reported experiencing some improvement in one or more PASC symptoms. For example, 72% of participants reported an overall improvement in their energy levels, 39% of participants reported improvement in “brain fog,” and 20% of participants reported improvement in sleep.

*“It helps with pain relief*,* and memory*,* brain fog. I think it helped with everything. … I felt better after each class and it has continued to help.” (ID 36)*.

*“I’ve been actually sleeping much*,* much better. Well*,* well-rested. I had an issue with lack of energy. That’s been restored. I feel energetic*,* you know*,* starting to get up at the time that I used to get up before COVID*,* which is around 5:30 or 6:00 in the morning*,* and now I’m back to that kind of routine. I had some issues with concentration and kind of a brain fog kind of a thing that has mostly gone away.” (ID 21)*.

Other PASC symptoms that participants reported improvement in were digestive symptoms, anosmia, arrhythmias, joint pain, headaches, and depression.

*“So*,* I was having arrhythmia several times a week. And it was debilitating. There were days I’d have to miss school and stuff because I could not walk. I couldn’t*,* I couldn’t hardly breathe. It was just miserable. Well*,* during the month of April*,* which I was in qigong*,* the entire time*,* I only had three episodes the entire month. I only had three episodes. Instead of multiple each week.” (ID 35)*.

*“I feel better*,* meaning like the joints weren’t so achy … I noticed my smell was coming back.” (ID 17)*.

*“I am a firm believer that it was the power of qigong*,* you know*,* that healing energy that helped my heart go from being in a rapid a-fib to normal sinus rhythm.” (ID 63)*.

*“My depression has been lessened. … I developed this rash with COVID … And I noticed on the last day of class when I looked out at my arm*,* the rash wasn’t there.” (ID 44)*.

Of the participants who experienced symptom improvement, the degree of improvement ranged from complete resolution of symptoms to mild relief. Some participants reported symptom improvement that only lasted temporarily, some noted improvement in some PASC symptoms while other symptoms remained unaffected, and some reported no change in their symptoms at all.

*“The POTS is 100% resolved. My oxygen is usually fluctuating anywhere between 96% and 100%. I no longer use my oxygen. I am back to work in the office full time. I’m no longer bedridden*,* I do not have pain*,* and my muscles do not give out*,* I’m dancing with my kids again*,* I’m out and about*,* I’m socializing*,* I’m in public*,* I essentially feel as though qigong gave me like my life back. … The people closest to me say that I got the sparkle back in my eyes*,* my energy levels are higher*,* I am more focused and in tune at work*,* I’m knocking stuff out faster. I just feel like I woke up from a horrible nightmare. Essentially*,* I feel like I’m myself again.” (ID 30)*.

*“I do feel like I have improved from like where I was at at the beginning. … I don’t feel like it cured my long COVID unfortunately*,* but I do think it helped.” (ID 9)*.

*“I didn’t feel bad after the session by any means*,* but I didn’t feel anything was changing. … I think it was good experience for me. I just*,* again*,* I just don’t know whether it helped me*,* to be determined.” (ID 11)*.

**Fig. 1:**
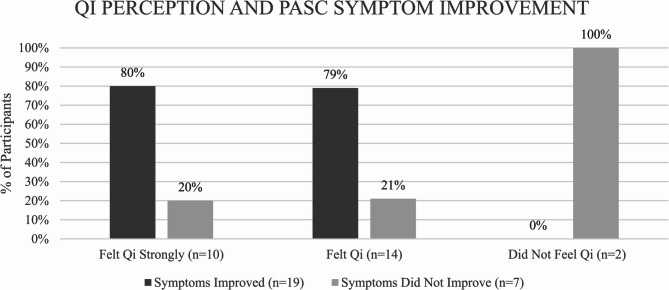
Qi Perception and PASC Symptom Improvement. Participants’ ability to feel qi and their experience of symptom improvement were based on descriptions provided in participant interviews. Symptom improvement was coded as sustained improvement in PASC symptoms that varied from partial to complete

Participants’ perceptions of qi were compared to improvement in PASC symptoms (Fig. 1). 79% of participants who perceived qi to some extent (*n* = 24) experienced improvement in PASC symptoms. Conversely, the two participants who reported no perception of qi did not experience any symptom improvement.

### Group dynamic

Almost every participant discussed positive aspects of the group experience in their interview. Participants described a sense of community that was formed in the study group, along with an understanding and validation of their experience living with PASC.

*“Getting to talk about what they were going through was actually just as helpful as whatever the qigong might have done*,* I think. … Knowing that other people were going through the same thing*,* what they were going through and sharing that*,* helps my mental.” (ID 8)*.

*“Not only did some of my symptoms alleviate and I was feeling better*,* but it was also like a group therapy at the same time and that was really helpful. … That we were in person*,* I think was key*,* and that we could all share our unfortunate journey with long COVID*,* and we relate to each other.” (ID 39)*.

A few participants shared the positive experience of meeting another person with PASC for the first time.

*“I never met somebody else with long COVID*,* because it’s been an isolated experience. And then coming to sessions week after week*,* having folks that have gone through similar symptoms and how they’re managing it … it was just really nice to be surrounded by a group of individuals who knew what we were going through.” (ID 50)*.

*“I just have a better understanding of what long COVID is*,* and I was very relieved to talk to someone else who was also suffering from long COVID.” (ID 20)*.

### Well-Being and a shift in mindset

Participants were asked, “Do you think the qigong sessions affected your symptoms or sense of well-being in any way? Please explain.” Of the 26 participants, 22 reported an improvement in their well-being. The remaining four participants gave a response to the question that was unclear to coders, but no participants definitively reported that their well-being did not improve. When explaining their response, participants described different aspects of their experience in the study and the practice of qigong that may have contributed to this improvement. 40% of participants reported an increased sense of calmness or relaxation, with some describing qigong as a meditative practice.

*“Just sort of like being forced to be there and being forced to like kind of just meditate in a chair for a couple minutes was like very calming.” (ID 16)*.

Some participants described how they found it beneficial to continue practicing qigong by themselves, or with others, in their daily lives.

*“And I go to it at least once a day*,* just as a practice now to try and really just for me*,* a source of like*,* like I said*,* it’s also kind of like a meditation type of a practice for me as well.“ (ID 6)*.

*“I’d wake up and I’d have like*,* a chest pain*,* which I get periodically*,* and I said*,* ‘Oh*,* you know what*,* I’m going to go outside and do the self-guided qigong’*,* and that helped. It actually helped. And so*,* I’ve used that several times.” (ID 36)*.

*“Because if I do this before bed*,* or I wake up during [the] night and can’t go back to sleep right away*,* if I do this at ten*,* this will let me go right back to sleep. … Definitely feel like I have a really valuable tool in my tool chest now. … Feeling enabled to have a non-invasive tool*,* and a free tool*,* you know*,* that I can do on my own to help myself and share with others who may be suffering from things like this.” (ID 57)*.

Separate from any symptom improvement participants experienced, they described an increase in mental/emotional resilience to PASC symptoms, as well as increased resilience to everyday stress.

*“Going through each week and noticing my energy improving*,* my resilience to stress improving*,* my stamina improving.” (ID 57)*.

*“So*,* now*,* what happened with my symptoms is they’re there*,* but I don’t – my reaction to them are not the same as they were. It’s like*,* ‘Oh yeah*,* hello friend*,* let’s have some qi’*,* you know*,* that kind of thing. … I’m more comfortable with what happened and what is happening with me*,* and I think I know that that makes the symptoms feel less. … They’re still there*,* but you know*,* my reactions to them are different.” (ID 23)*.

*“My symptoms are still here*,* but they don’t bother me as much*,* or like I am not*,* I don’t suffer from them quite as much*,* like my attitude about them is different*,* if that makes sense. And*,* like I still get winded very easily*,* you know*,* like if I bend down to*,* like*,* pick something up off the floor and stand back up*,* … I lose my breath*,* like I’ve just ran a marathon*,* you know? And that still happens to me. But it’s not as distressing as it was before*,* like*,* I don’t know how else to describe it. … Like*,* my recovery from it is easier*,* I still deal with insomnia*,* but I*,* you know*,* my attitude after a sleepless night is not so grim. I guess … my general outlook on everything is better.” (ID 58)*.

Participants also described an increase in hope and positivity as a result of the study.

*“I think our attitude about anything that’s going on with our body is real important. And this gave me*,* this study gave me acknowledgement*,* it gave me hope*,* and it gave me something that I can work with.” (ID 23)*.

*“I think it gave me more of a positive outlook.” (ID 43)*.

*“And this is the first time*,* you know*,* when I could actually see like*,* being well as a real possibility. Like I’m not there yet*,* but it actually feels like it could be like a real possibility and just having that kind of hope is kind of*,* it’s life changing to actually think that that’s a real possibility. Instead of*,* you know*,* like ‘Oh God*,* this is how I have to live forever and ever’. And that kind of doom that comes with it*,* I don’t feel that.” (ID 58)*.

## Discussion

Nested within a pilot randomized controlled trial examining qigong for PASC, we present the first qualitative study of combined external and internal qigong as a treatment for a medical condition. This study explored how patients with PASC experienced and understood qi and examined the patient perspective on how group-based qigong practice impacts PASC symptoms and overall well-being.

Most participants were naive to qigong, yet nearly all of them were able to perceive qi, a novel finding that has not yet been described in the limited qualitative research on qigong [24, 25]. Participants described external qigong in a variety of ways, including depictions of energy movement, the promotion of healing, and a calming and restful experience. A few participants had difficulty describing this form of energy healing. Participants in this study described their experiences of qi similarly to descriptions in studies on other forms of biofield therapies. In qualitative studies on therapeutic touch, Reiki, pranic healing, and Magdalena energy healing, participants reported experiences of temperature and body sensations, increased awareness, relaxation, symptom changes, post-session drowsiness, and an overall positive experience of the treatment [26–29].

In this study, 73% of participants reported some degree of symptom improvement and 85% an increase in their overall well-being. Two study participants were unable to perceive qi and also noted no improvement in symptoms. It is unclear whether the ability to perceive qi is necessary to experience benefit from this therapy, though our data suggests that qi perception itself may not be sufficient.

The positive effect of the intervention on symptoms ranging across body systems and on overall well-being is a finding mirrored in other qualitative studies on other biofield therapies. Interestingly, 40% of participants in our study reported an increased sense of calmness or relaxation, while 68% of participants reported experiencing calmness or relaxation in a qualitative study on the subjective experience of Reiki [27]. A qualitative study of energy healing for patients with irritable bowel syndrome and inflammatory bowel disease yielded mixed results with some participants reporting improvements in symptoms, while others noted no change [26]. A qualitative study of reiki in hospital nurses demonstrated an overall positive effect on sleep quality, though a few participants did not experience any improvement [30]. Other studies on biofield therapies have similarly demonstrated improvements in sleep quality, physical symptoms, and psychological well-being [27, 29, 31]. Participants in two published qualitative studies on internal qigong (for cancer survivors and patients with chronic medical conditions), described improvements in physical and mental health, well-being, and social relationships [24, 25].

In this study, participants affirmed that the delivery of the intervention in a group setting contributed to their positive experiences, citing a sense of community, validation, and sharing of ideas for symptom management. Group-based interventions have demonstrated statistically significant improvements in pain, self-efficacy, self-care, psychological outcomes, and quality of life for patients with a variety of long-term conditions [32]. Thus, the group setting in which the qigong intervention was delivered may have contributed to some of the positive outcomes observed. The generally positive effects of group-based interventions, and the relative isolation that many individuals with PASC feel [33], suggest that group-based delivery of interventions for this patient population may be particularly helpful and effective.

Limitations in the design of this pilot feasibility study include the small sample size, use of a single qigong instructor, and the multimodal nature of the intervention that makes it impossible to attribute the effects observed to any particular component (i.e., external qigong, internal qigong self-practice, and the group-based nature of the intervention). It is also possible that the intervention components interacted synergistically to facilitate participants’ improved well-being. For example, the internal qigong self-practice may have sensitized participants to benefit more from external qigong or vice versa. Variations in the descriptions of qi may, in part, be due to whether participants were referring to their experience of the external vs. internal qigong practice. Another potential limitation may be bias in that only participants who were open to receiving external qigong were included in the study. Our results may hold more positive views on the intervention for that reason. However, most participants knew little about external qigong, or reported being skeptical, prior to beginning the study. Indeed, witnessing others’ experiences of the qigong intervention may have made some participants more open to exploring it. Strengths include achievement of data saturation in our qualitative analysis. Additionally, all participants who completed the study were interviewed, and study staff who conducted the interviews and analyzed the transcripts were not involved in the intervention itself, with one of the coders abstaining from conducting interviews. Finally, the interview questions did not probe about changes in specific PASC symptoms, so our estimates of symptom-specific improvement may, in fact, be underestimates. Future studies should consider inclusion of a larger sample size with increased racial and ethnic diversity, the use of multiple qigong practitioners, and potentially blinding of participants to the intervention and/or testing individual components of the intervention (e.g., external vs. internal qigong vs. their combination).

## Conclusions

This study is one of few qualitative studies of qigong as a treatment for a medical condition. It presents a unique patient perspective on the effects of qigong, as well as insight into participants’ perception and understanding of qi. Most participants were able to perceive qi. The positive preliminary findings of this study warrant further research to evaluate the potential benefits of qigong in this patient population.

## Data Availability

De-identified data are available from the corresponding author upon reasonable request.

## Abbreviations

PASC: Post-Acute Sequelae of COVID-19
TCM: Traditional Chinese Medicine
FVC: Forced vital capacity
SD: Standard deviation
